# Machine learning methods to predict the cultivation age of Panacis Quinquefolii Radix

**DOI:** 10.1186/s13020-021-00511-5

**Published:** 2021-10-09

**Authors:** Xiaowen Hu, Hua Yan, Xiaodong Wang, Zonghu Wang, Yuanpeng Li, Lianjun Zheng, Jianbo Yang, Wenguang Jing, Xianlong Cheng, Feng Wei, Shuangcheng Ma

**Affiliations:** 1 National Institutes for Food and Drug Control, Institute for Control of Chinese Traditional Medicine and Ethnic Medicine, Beijing, 100050 China; 2 XtalPi-AI Research Center (XARC), Tower A, Dongsheng Building, No. 8, Zhongguancun East Road, Haidian District, Beijing, 100083 China

**Keywords:** Applicability domain, Cultivation age, Machine learning, *Panax quinquefolium* L.

## Abstract

**Background:**

American ginseng (AG) is a valuable medicine widely consumed as a herbal remedy throughout the world. Huge price difference among AG with different growth years leads to intentional adulteration for higher profits. Thus, developing reliable approaches to authenticate the cultivation ages of AG products is of great use in preventing age falsification.

**Methods:**

A total of 106 batches of AG samples along with their 9 physicochemical features were collected and measured from experiments, which was then split into a training set and two test sets (test set 1 and 2) according to the cultivation regions. Principle component analysis (PCA) was carried out to examine the distribution of the three data sets. Four machine learning (ML) algorithms, namely elastic net, k-nearest neighbors, support vector machine and multi-layer perception (MLP) were employed to construct predictive models using the features as inputs and their growth years as outputs. In addition, a similarity-based applicability domain (AD) was defined for these models to ensure the reliability of the predictive results for AG samples produced in different regions.

**Results:**

A positive correlation was observed between the several features and the growth years. PCA revealed diverse distributions among different cultivation regions. The most accurate model derived from MLP shows good prediction power for the fivefold cross validation and the test set 1 with mean square error (MSE) of 0.017 and 0.016 respectively, but a higher MSE value of 1.260 for the test set 2. After applying the AD, all models showed much lower prediction errors for the test samples within AD (IDs) than those outside the AD (ODs). MLP remains the best predictive model with an MSE value of 0.030 for the IDs.

**Conclusion:**

Cultivation years have a close relationship with bioactive components of AG. The constructed models and AD are also able to predict the cultivation years and discriminate samples that have inaccurate prediction results. The AD-equipped models used in this study provide useful tools for determining the age of AG in the market and are freely available at https://github.com/dreadlesss/Panax_age_predictor.

**Supplementary Information:**

The online version contains supplementary material available at 10.1186/s13020-021-00511-5.

## Background

*Panax quinquefolium* L., also known as American ginseng (AG), is a perennial herb native to Canada and the eastern United States. It is widely consumed as herbal medicine throughout the world for a variety of medicinal benefits including antioxidant [1], neuroprotective [2], anti-cancer [3], and improving neurocognitive function [4]. The biological activities are mainly derived from the ginsenosides constituents in AG among other active ingredients like polysaccharides and peptides. AG has a higher content of ginsenosides compared to its family member *Panax ginseng* (PG), another major cultivar of ginseng [5]. AG also features a calm effect, or “yin” property according to traditional Chinese medicine theory [6, 7], and thus is applicable to much wider populations than PG, which contains a stimulant and has a “warm” or “heat” property [8, 9]. These properties lead to the prevalence of AG products like herbal teas and AG wines in China, as well as the usage of AG as a major ingredient in some Chinese patent medicines, such as Xinyue capsule and Fufang Zaofan Pill recorded in the official Chinese Pharmacopoeia [10].

Owing to a growing demand for health products and tonics, AG was introduced to China early in 1975, and now are extensively cultivated in Beijing, Jilin, Liaoning, and many other areas. It has long been a problem to fairly evaluate the grade of AG products and to determine their prices in the commercial market [11]. Usually, the prices of AG in the market are decided in an empirical and subjective manner according to their morphological properties, growing regions, cultivation ages, etc. [8, 12]. Among these factors, the cultivation age is one of the most convincing judging criterion for grades and prices determination as it largely affects the accumulation of bioactive compounds. As a result, the market price varies widely among AG with different cultivation years [11, 13]. This leads to the sellers pass off younger AG with a cheaper price as an older AG for higher profits. Therefore, it is crucial to develop a reliable method to authenticate the growth year of AG to combat age falsification.

Traditionally, AG can be aged through the number of prongs or the number of stem vestiges before harvest [14], but it is not applicable to AG products in the market that usually lose these morphological characteristics. Modern analytical techniques (e.g., NMR, HPLC) have enabled quantitative analysis of active ingredients in medicinal herbs. By establishing a relationship between metabolite fingerprints and the growth year statistically, predictive models can be built to help estimate the age of plants [12]. For example, an NMR-based metabolomics technique was used to determine NMR spectrum of 70 ginseng root samples of 2-, 3-, 4-, and 5/6-years old, then partial least squares (PLS) model was employed and trained, achieving a root mean square error of prediction (RMSEP) value of 0.2 on the test set [13]. Similar models for rhizome age prediction were developed using IR spectroscopy combined with PLS regression, and the RMSEP value can be as low as 0.036 [15]. Other statistical analysis methods including principal component analysis (PCA), hierarchical cluster analysis, and linear discriminant analysis could also discriminate the growth years of ginseng roots by their chemical profiles measured by other analytical methods [16–18]. However, most of the statistical analyses implemented in the above studies are linear in nature, which are unable to fully capture the intrinsic relationships between physicochemical profiles and growth year, and would easily fail for complex prediction tasks (we will compare the performance of linear and non-linear models later). Moreover, due to the relatively small datasets employed and a lack of applicability domain (AD) definition, it is hard to evaluate to what extent the predictive results on new samples would be statistically sound and reliable.

Machine learning (ML) is a well-developed discipline that has been used in numerous fields, such as ADMET prediction [19], ligand-based virtual screening [20], as well as the plant science [21]. It can identify an inherent pattern from the existing data through diverse algorithms and predict the new data with high portability. In this study, we explore how ML methods can be employed to accurately predict the growth year of AG using physicochemical profiles collected from experiments on a large data set containing 106 samples. We also seek to reduce the uncertainty in prediction by developing AD for the models. Ultimately, we will show that ML models regulated by AD have a satisfactory predictive power. These methods and results provide useful guidance for the identification of AG ages in the market.

## Materials and methods

### Materials

Five ginsenoside standards were purchased from National Institutes for Food and Drug Control (Beijing, China). HPLC-grade acetonitrile was purchased from Fisher (USA). Purified water was prepared using a Milli-Q purification system (Millipore, USA). Other chemicals were of analytical purity.

A total of 106 cultivated AG samples with ages ranging from 2 to 4 years were purchased from Kangmei Pharmaceutical Co., Ltd., which originated from 5 different areas of Jilin, Liaoning, Shandong, Beijing, and Shanxi provinces. These samples were harvested from the planting bases, rinsed in water and air-dried locally according to the Good Manufacturing Practice (GMP). The cultivation ages were provided by the planting bases and the reliability of the cultivation age could be guaranteed. All herbal medicines were authenticated by Professor Hua Yan. The voucher was deposited in the Institute for Control of Chinese Traditional Medicine and Ethnic Medicine (Beijing, China).

### Preparation of standard and sample solutions

Each sample was levigated into powder and sieved through a 60-mesh screens. One gram of the pulverized AG and 25 ml methanol was weighed accurately and transferred to a 50 ml conical flask. The suspension was maintained for 1 h followed by ultrasound-assisted extraction for 30 min at room temperature. After additional methanol was added to restore its original weight, the extract was fully blended and the supernatant was filtered through a 0.45 μm membrane filter, which will be used later as test solution. 20 μl of the filtrate was subjected to HPLC for analysis.

Ginsenoside standards Rg1, Re, Rb1, Rd and pseudo F11 were prepared separately in a similar way to yield stock solution of 0.5 mg/ml in methanol. 2 ml of each reference solution were combined and diluted to 25 ml, which was then filtered through a 0.45 mm filter membrane for retention time analysis.

### HPLC conditions

HPLC was performed on a Waters 2695–2996 instrument equipped with a diode-array detector, automatic injector, thermostatically controlled column compartment, an online degasser, and an Alltech ES2000 evaporative light scattering detector (ELSD). Separation was accomplished using a Grace Vydac 208TP C8 column (250 × 4.6 mm, 5 μm) at 30 °C. The detection wavelength of the UV absorption spectrum was set to 203 nm. The mobile phase was composed of CH_3_CN (A) and H_2_O (B). The gradient elution program with 1 ml/min flow rate was used as follows: 0–10 min, 20% A; 10–11 min, 25% A; 11–33 min, 33% A; 33–38 min, 46% A; 38–40 min, 80% A; 40–45 min, 100% A; 45–55 min returned to 20% A. The drift tube temperature and the nitrogen gas flow rate of ELSD were set to 110 °C and 2.5 l/min, respectively.

### Calibration curve of ginsenoside standards

A series of reference mixtures containing Rg1, Re, Rb1, Rd, and pseudo F11 were obtained by diluting the stock solutions and injected in triplicate into HPLC. The standard curves of Rg1, Re, Rb1 and Rd were constructed based on the areas of the UV absorption peak in the chromatograph (dependent variable) and the corresponding amounts of the analyte (independent variable), while the calibration curve of pseudo F11 was built using the area values in the ELSD chromatograph. The content of five saponins in the sample was calculated using the corresponding calibration curves.

### Determination of ethanol and aqueous extractives

The weight percent of ethanol extractives was determined by hot extraction method. The content of aqueous extractives was calculated using cool extraction method. Both the two methods are performed according to the Chinese Pharmacopoeia [10].

### Data preparation

To conduct a comprehensive assessment of ML models, the 106 AG samples were divided into a training set and two external test sets based on their cultivation regions. The training set was used to optimize the hyperparameters of the models and consisted of 64 samples (2–4 years old) collected from Jilin, Liaoning, and Shandong provinces. The two external test sets were used to evaluate the performance and generalizability of the trained models, and were made up of 42 samples (4 years old) originating from Beijing (test set 1, 25 samples) and Shanxi (test set 2, 17 samples) province, respectively.

A total of nine features were used to represent each sample. Seven of them were chemical properties including the content of five saponins (Rg1, Re, Rb1, Rd and F11) and the content of alcohol and aqueous extractives as described above. The other two features were the length and weight of each sample that represent the physical profile, where the lengths of the AG were approximate values since the tap root and the fiber root in most of the samples were truncated. All data used in this study are available in Additional file 1: Table S1.

### Feature distribution of the collected data sets

PCA was carried out to compare the distribution of the training set and two external test sets. All features were first standardized by subtracting the mean value and divided by the standard deviation. After dimensionality reduction, most of the information in the original descriptors was compressed into the first three principal components (PCs), which were plotted as scatters in a three-dimensional (3D) space and projected into three two-dimensional (2D) planes to compare the diversity of the three data sets intuitively.

### Model building

To obtain a robust and effective model, four machine learning algorithms including elastic net (EN), k-nearest neighbors (KNN), support vector machine (SVM), and multi-layer perceptron (MLP) were selected and to construct predictive models. Hyperparameter optimization was performed for all models by using a grid search strategy with fivefold cross-validation. The implementation was conducted using Python (ver. 3.8.10) and the scikit-learn library (ver. 0.24.1).

EN is a regulated linear regression model. As opposed to multivariate linear regression, two penalty terms, *L*_1_ and *L*_2_, were added to the loss function of EN to reduce model complexity. These penalties were tuned by alpha (*λ*_1_ + *λ*_2_) and l_1__ratio [*λ*_1_/(*λ*_1_ + *λ*_2_)] in the ElasticNet function, where *λ*_1_ and *λ*_2_ are the weights of *L*_1_ and *L*_2_ regularization, respectively.

KNN regression is a conceptually simple yet useful method based on feature similarity [22]. Two steps are required to predict the label of a query sample. First, the distances between the query sample and the samples in the training set are computed to determine its *k* nearest neighbors. Then the mean or median value of the labels of these neighbors was taken to be the final prediction.

Support vector regression (SVR) is a branch of the SVM. Unlike most regression algorithms that aim to minimize the prediction error, the goal of SVR is to minimize the generalization error bound which is determined by a subset of training data called support vectors. In addition, kernel technique can be applied to handle nonlinear tasks. Herein, radial basis function (RBF) was used as the kernel function. Two parameters, the regularization parameter *C* and the kernel coefficient gamma, were tuned to obtain the optimal model [23].

MLP is a class of feedforward neural networks composed of three types of layers: an input layer, one or more hidden layers, and an output layer [24]. Each layer possesses several neurons and is connected with each other through layers of nodes. The neurons in the hidden layer are activation functions applied to the sum of weighted inputs and propagate the results to nodes in the next layer. The training process was to update the connection weights iteratively by backpropagation. In this work, the neurons in the hidden layers adopted tanh as the activation function. The regularization term alpha, size of the hidden layer, and learning rate were optimized for better model performance.

### Model performance evaluation

Two statistical metrics, coefficient of determination (*R*^2^, Eq. 1) and mean squared error (*MSE*, Eq. 2), were computed to evaluate and compare the performance of the four regression models:

1 $$R^{2} = 1 - \frac{{\mathop \sum \nolimits_{i = 1}^{n} \left( {y_{i}^{pred} - y_{i}^{true} } \right)^{2} }}{{\mathop \sum \nolimits_{i = 1}^{n} \left( {y_{i}^{pred} - y_{i}^{true\;mean} } \right)^{2} }},$$

2 $$MSE = \frac{{\mathop \sum \nolimits_{i = 1}^{n} \left( {y_{i}^{pred} - y_{i}^{true} } \right)^{2} }}{n},$$

where $y_{i}^{pred}$, $y_{i}^{true}$, $y_{i}^{true\;mean}$ are predicted, actual, and mean value of the growth years of AG, respectively. *R*^2^ measures the proportion of the total variance in dependent variable (*Y*) that is explained by the independent variable (*X*), and was used in cross-validation and model optimization. MSE was used to evaluate the average of the square of the error between actual and estimated values, and was used for model evaluation on the test sets.

### Determination of applicability domain

Usually, the training set only represents a small subset of the whole feature space because of the limitation of the training samples. For samples that differ substantially from the samples in the training set, the prediction results would be highly unreliable and thus should be excluded. In this regard, applicability domain of a model can be defined to estimate whether a new sample is similar enough to the training samples. TAD (Eq. 3) was used here as the threshold of AD:

3 $$TAD = \frac{1}{n}\mathop \sum \limits_{i = 1}^{n} \overline{d}_{ki} + Z\sigma ,$$

where $\overline{d}_{ki}$ is the average of Euclidean distances between the training data *i* and its *k* nearest neighbors in the training set, *σ* is the standard deviation of the average distances aforementioned, *Z* is an empirical parameter [22, 25, 26]. When a query sample *j* comes in, the threshold of sample (TS, Eq. 4) can be computed and compared to TAD to decide whether the query is within the AD:

4 $$TS_{j} = \overline{d}_{kj} ,$$

where $\overline{d}_{kj}$ is similar with the $\overline{d}_{ki}$ expect for using query data *j* instead of training data *i*. If TS > TAD, i.e., the sample was far from the *k* nearest neighbor points, the predictive result may be marked as unreliable, and vice versa. The *k* and *Z* values determine how strict the AD is defined. For example, as *Z* decreases, the TAD value declines and the AD shrinks, meaning fewer data points are expected to be reliable. The impact of *k* and *Z* were investigated by estimating prediction errors of test samples within the AD. *Z* values were increased from 0.5 to 3 with step 0.2 and *k* varied from 1 to 12.

## Results

### Ginsenoside profiles of the AG samples

Five saponins in the HPLC chromatograms were assigned unambiguously by comparing with the reference standards and their retention time. Standard curves were built and their correlation coefficient values (*R*^2^) were calculated from the concentrations and peak areas for each sample. All calibration curves shown a good linearity with *R*^2^ > 0.99 (Additional file 1: Table S2). The contents of the 5 saponin monomers in the 106 AG samples were calculated using the standard curves. The nine physicochemical features are listed in Additional file 1: Table S1.

**Fig. 1:**
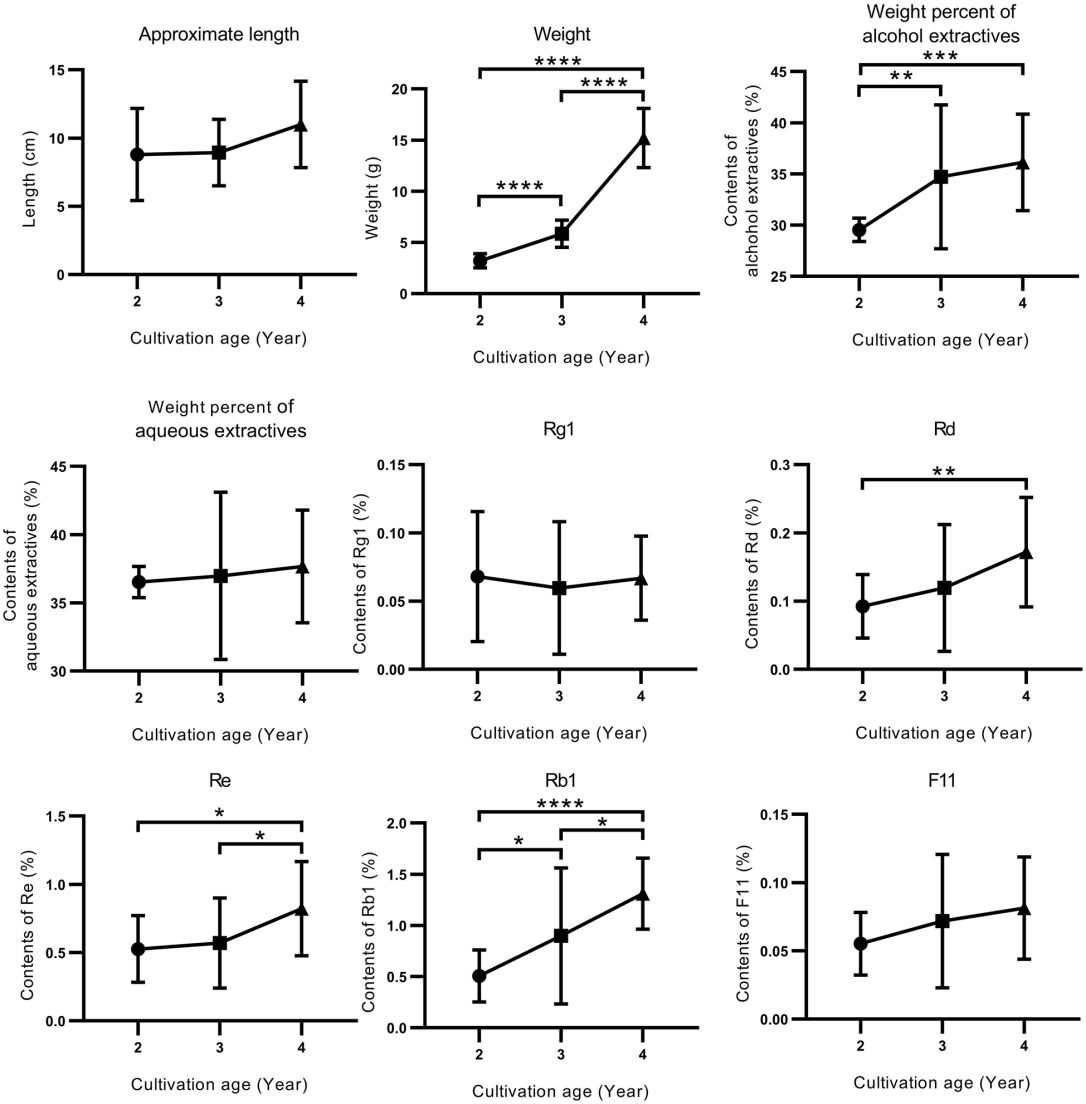
The correlation between cultivation age and 9 physicochemical features for the samples in the training set. Data are expressed as mean ± SD. *P < 0.05, **P < 0.01, ***P < 0.001, ****P < 0.0001, by two tailed Student’s t test

In general, the physicochemical features are largely affected by the growth year and cultivation regions. For the samples in the training set, most of the features positively correlated with growth years (Fig. 1). Especially the weight and the major constituent Rb1 varied remarkably in different ages. The contents of ginsenoside Rd, Re and the weight percent of alcohol extractives also increased with the growth year. While other properties fluctuated slightly within a certain range.

Cultivation region also greatly affects the physicochemical profiles, as most features including length, weight, contents of alcohol and water extractives are different for samples that are of the same age but from different producing areas (Additional file 1: Figure S1). The content of aqueous and alcohol extractives in the AG samples from Shanxi is the highest, followed by Jilin, and Beijing. Reversely, the morphological features of samples from Shanxi are the lowest while the largest AG was cultivated in Jilin with the average weight of 15.2 g and 11.0 cm in length. Furthermore, the samples from Beijing yield the highest level of Rg1 and the lowest level of Rb1, resulting in a lowest Rb1/Rg1 ratio among the three regions, almost two times lower than those from Jilin and Shanxi. The variation in saponins content may result in different bioactivities of the final AG product as demonstrated by the previous studies [7]. The dependence of ginsenoside contents in AG on either growth year or producing region we observed here is consistent with previous findings [9, 15, 27, 28].

### Feature distributions of the training set and the test sets

**Fig. 2:**
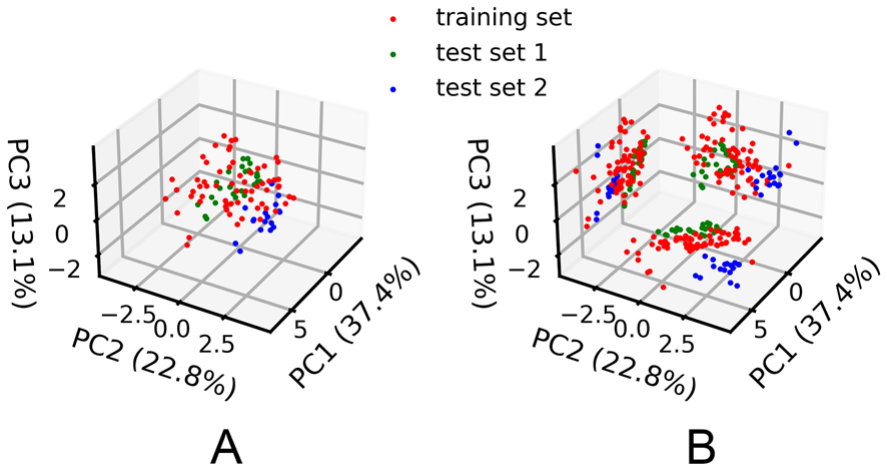
Spatial distributions of the training set (red dots), test set 1 (green dots) and test set 2 (blue dots) after applying PCA. **A** Scatter plot in 3D space; **B** projection on three 2D planes. Feature space of test set 1 partially overlaps with that of the training set, whereas most samples in test set 2 fall outside the feature space of the training set

PCA scores plot was used to visualize and rationalize spatial distribution of the training set and the test sets. After feature decomposition, the first three PCs account for 77.3% of the total variance. The distributions of the training set and two test sets on the three PCs are displayed in Fig. 2.

The feature space of test set 1 is partially overlapped with that of the training set, and tends to gather toward the edge of the feature space of the training data, whereas test set 2 lies even further from the training set than test set 1. The data points in the training set are mostly distributed between − 3 to 2 on PC2, while the features of test set 2 are chiefly distributed between 2 to 4 on PC2 as an isolated cluster. The feature distributions of the training set and test set 1 shares some similarities in general, indicating that the samples from Beijing province are suitable for the evaluation of the constructed models. In contrast, the feature space of test set 2 (samples collected from Shanxi) differs distinctly from that of the other two data sets, and thus it is expected that the prediction results of test set 2 would be inaccurate. Although the two test sets show varying distribution compared to the training set, these test sets are helpful to establish an appropriate AD for the predictive models, which will be described in the next sections.

### Cross-validation and performance evaluation of the four regression models

**Fig. 3:**
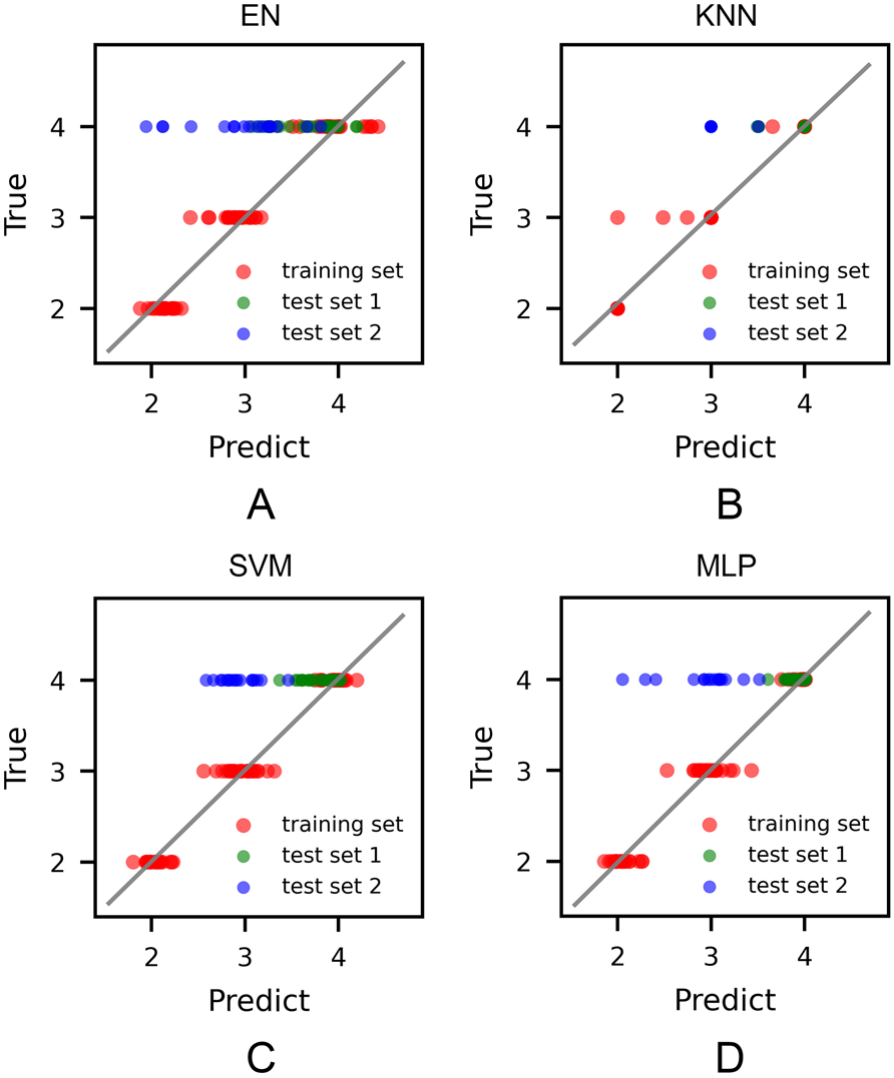
Scatter plot of the true ages and predicted ages of four ML algorithms. The regression line is colored in gray. The red dots represent the predicted results of fivefold cross validation of the training set. The green and blue dots represent the predicted values of the test set 1 and test set 2, respectively

Four regression models (EN, KNN, SVM, MLP) were trained and optimized on the training set consisting of 64 samples with fivefold cross validation. The generalizability of the models to independent data set was measured on two test sets consisting of 25 and 17 samples, respectively. Results were summarized in Table 1 and depicted in Fig. 3.

**Table 1:** Performance of the four regression models on the training set and the two test sets

Method	Fivefold cross validation		Test set 1	Test set 2
	*R*^2^	MSE	MSE	MSE
EN	0.912	0.042	0.243	1.370
KNN	0.954	0.022	0.070	0.956
SVM	0.963	0.019	0.070	1.180
MLP	0.965	0.017	0.016	1.260

For the cross-validation, all of the four ML algorithms, especially MLP, achieve good fitness with *R*^2^ > 0.9. MLP outperforms the other four models with the highest *R*^2^ value of 0.965 and the lowest MSE value of 0.017, closely followed by SVM and KNN with an *R*^2^ value of 0.963 and 0.954, and an MSE value of 0.019 and 0.022, respectively. EN has a slightly higher MSE of 0.042 and a lower *R*^2^ of 0.912, but is fairly acceptable.

The feature importance was measured by calculating the permutation importance of four ML models (Additional file 1: Table S2). The weight of the samples shows the highest importance for all of the models, followed by the ethanol-soluble extractives, aqueous soluble extractives, and the length of AG. Whereas the importance level of five saponin monomers was lower compared to other parameters. These results suggest that chemical features based solely on the saponin monomers are insufficient for the statistical models. More representative features such as ethanol-soluble extractives that can reflect the overall characteristics are essential to improve the precision of ML models.

Similar trends are observed on the test set 1. MLP shows good prediction accuracy on test set 1 with MSE values of 0.016. SVM and KNN also have a moderate MSE value of ~ 0.070, whereas EN has the largest MSE value of 0.243. A larger overall prediction error on the test set 1 can be expected since the feature space of test set 1 is not fully covered by that of the training set (Fig. 2). In such case, MLP still presents similar MSE values with the cross-validation ones, showing better generalizability. As expected, all the models fail to correctly predict the cultivation ages of AG samples in the test set 2 due to the distinct data distributions of the training set and test set 2 shown by the PCA results.

In summary, for the training set and test set 1, MLP achieves superior performance and generalizability than SVM, KNN, and especially EN, indicating that linear models might not be adequate for accurate prediction of AG cultivation years built on physicochemical features. As a result of a unique feature distribution, these models fail to predict the growth years accurately on the test set 2. But this reminds us that when the models are applied to samples whose producing regions are unknown, it is crucial to assess whether the samples are within the definition of the models instead of blindly accepting the prediction results. We will define ADs for our models to help provide confidence for the prediction results in the next section.

### Applicability domain definition

**Fig. 4:**
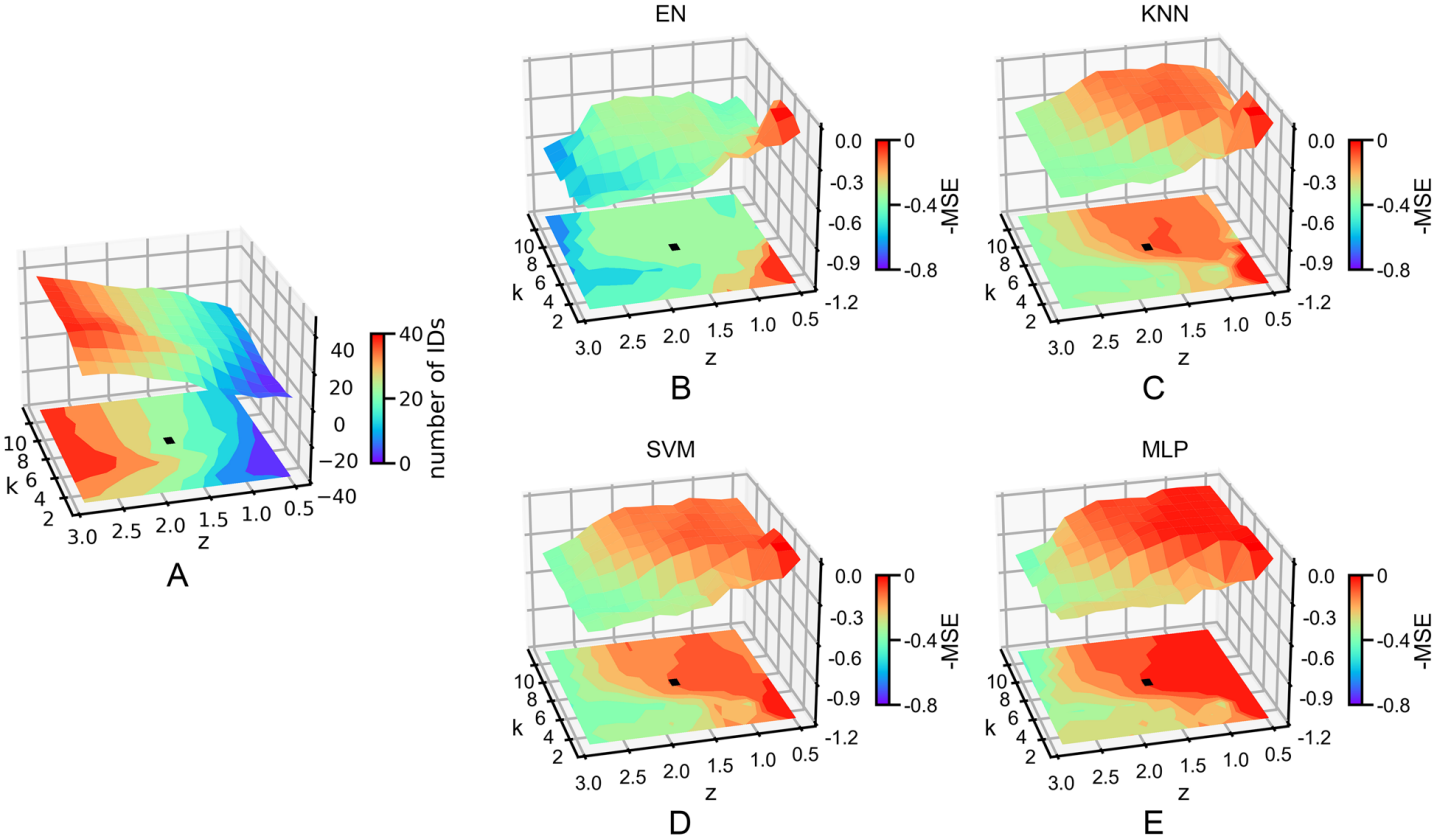
3D surface plots of **A** the number of samples in the test sets that fall within the AD (IDs) as a function of *k* and *Z*; and **B**–**E** the negative value of MSE predicted by the four ML models for the test samples within the AD as a function of *k* and *Z*. *k* = 6 and *Z* = 1.6 (black dot in each figure) are chosen to define the AD for all models

An AD based on feature similarity is defined for the four regression models. Optimal *k* and *Z* values for an appropriate AD definition are obtained by balancing prediction accuracy and the size of applicability range, which are monitored by the MSE value and the number of test set samples within the AD as a function of *k* and *Z* (see Fig. 4).

Figure 4A shows that the value of TAD increases with *k* and *Z*, and *Z* has a larger influence on TAD than *k*. At a small TAD, all models have very high accuracy, since very few test samples that closely resemble the training set are included. As the TAD becomes larger, the accuracy of the EN, KNN and SVM models decreases sharply then plateaus out, while the MLP model continues to have relatively smaller MSE (Fig. 4B–E). This plateau region is considered ideal for models to make stable and reliable predictions. When *k* and *Z* values are very large, much more samples are allowed for prediction, but the MSE significantly increases and the results are no longer trustable.

Finally, *k* = 6 and *Z* = 1.6 (black dots as shown in Fig. 4) were selected to define an AD that ensures higher confidence at the expense of a more restricted TAD. In this regime, 80% of the samples in test set 1 pass the TAD criterion, while only 1 out of the 17 samples in test set 2 is within the AD. Table 2 updates the MSE results in Table 1 by separating samples that are inside or outside of the AD (referred to as ID and OD, respectively). Among the four models, MLP exhibits an outstanding predictive power with an MSE value of 0.030. MLP, KNN, and SVM have a better performance on the ID samples than the EN, consistent with the trend in Table 1. Overall, the prediction errors of the OD samples are way above those of ID samples, showing that the AD established here can discriminate samples that are expected to have very inaccurate prediction results.

**Table 2:** Performance of the models on test samples that fall inside (ID) and outside (OD) the AD defined by *k* = 6 and *Z* = 1.6

Models	MSE of the test set 1		MSE of the test set 2		MSE of the two test sets	
	ID (20)	OD (5)	ID (1)	OD (16)	ID (21)	OD (21)
EN	0.288	0.063	0.658	1.414	0.306	1.093
KNN	0.087	0.000	0.246	1.000	0.095	0.762
SVM	0.082	0.020	0.288	1.236	0.092	0.946
MLP	0.020	0.000	0.233	1.324	0.030	1.009

The number of ID or OD samples is given in parentheses

## Discussion

AG has wide applications in pharmaceutical, nutraceutical, food, and cosmetic industries owing to its bioactive compounds such as ginsenosides and polysaccharides [29]. Commercial AG is priced mainly depending on the cultivation age, which is an important factor for the accumulation of bioactive compounds. Thus, the development of a convincing method to authenticate the growth year of AG is of great importance to prevent adulteration.

To this end, 106 batches of AG samples were collected as a data set. A preliminary analysis of 9 physicochemical features measured experimentally revealed that most features positively correlated with the growth year. That is, variation in cultivation age has a huge impact on the constituents, which may result in different bioactivities of the final AG product. However, intrinsic relations between cultivation age and chemical profiles can be a good pointcut for the construction of the model. Apart from the growth year, producing region is another factor that affects the content of active ingredients in AG, and should be paid special attention in the future when evaluating and using predictive models for cultivation age estimation.

Then the whole data set was split according to their cultivation regions for training and validation. The reasoning for choosing test sets based on cultivation regions instead of data distributions is as follows. Typically, stratified sampling and random sampling methods are adopted for train-test split to obtain a test set that has a similar data distribution to that of the training set. This works well when the distribution of the training/test set represents the real-world scenarios [30]. In our case, however, it is highly possible that the predictive models will be applied for AG products from cultivation areas that are very different from what we have included in the training set. Therefore, the generalizability of our models needs to be properly evaluated by using test sets that are collected from varied regions and have different feature distributions. These heterogeneous data were also useful for the rigorous construction of AD.

After optimization of the model, MLP shows the highest performance both in the training set and the test set 1 with MSE values lower than 0.018. While the EN has the least predictive ability. The poor prediction power of EN indicates that the relationship between the contents of active ingredients in AG and the cultivation ages is far from linear, and non-linear models should be considered in an attempt to improve the accuracy of predictive models on AG cultivation ages. Comparatively, MLP is more robust by having neurons with non-linear activation functions and high connectivity between layers, and would be more suitable for such complex problems.

Concerning the test set 2 (produced in Shanxi), all the models had high prediction bias, probably owing to the different feature patterns of AG samples in the test set 2. Therefore, an AD was customized to the models, and the defined AD could rule out the samples with higher errors. The results highlight the necessity for a rigorous definition of AD in the predictive model to ensure the reliability for the prediction of unknown AG samples. Besides, more representative samples originated from major cultivation regions need to be collected in the follow-up research for model construction in order to be more broadly applicable.

## Conclusion

In the present study, in silico models derived from four ML algorithms (EN, KNN, SVM, MLP) were constructed to predict the growth year of AG based on a curated data set consisting of 106 samples. Nine physicochemical features including the length and weight of the sample, and the content of 7 chemical constituents of AG were measured by experiments and used as inputs of the predictive models. Two external test sets consisting of AG samples produced in different regions were used to evaluate the generalizability of the models. Furthermore, a methodology based on Euclidean Distance was developed and proved to be feasible to estimate the uncertainty of the predictive results for all models. Most models achieved good accuracy on AG age prediction, especially MLP with an MSE of 0.030 on the test data within the AD. These results indicate that the ML methods have great potential for the prevention of age falsification and regulation of the Chinese medicine market.

In conclusion, we developed an effective approach for the accurate prediction of the AG cultivation ages. This is the first report on the AG cultivation age prediction by using AD-equipped ML methods. Particularly, all models and data used in this study are open access encouraging further analysis on the feature-label relationship and model training for tasks not limited to AG age authentication.

## Supplementary Information

**Additional file 1: Table S1.** Samples information and nine physicochemical features. **Table S2.** Calibration curves of the five saponins. **Figure S1.** Comparison of 9 physicochemical features among 4-year-old AG cultivated in Jilin, Beijing, and Shanxi. **Figure S2.** Measure of feature importance. (A–D) The permutation importance of EN, KNN, SVM and MLP. The code is freely available at https://github.com/dreadlesss/Panax_age_predictor.

## Data Availability

The datasets used in the current study are included in Additional file 1.

## Abbreviations

AG: American ginseng
RMSEP: Root mean square error of prediction
PCA: Principal component analysis
EN: Elastic net
KNN: K-nearest neighbors
RF: Random forest
SVM: Support vector machine
*R*^2^: Correlation coefficient
MSE: Mean square error
AD: Applicability domain
TAD: Threshold of the applicability domain
TS: Threshold of the sample
