# Supplementary material for: Machine learning methods to predict the cultivation age of Panacis Quinquefolii Radix
Source: Chin Med. 2021 Oct 9;16:100. doi: 10.1186/s13020-021-00511-5 (PMC8501543; doi:10.1186/s13020-021-00511-5)
Supplement: Supplementary file 1 — Additional file 1: Table S1. Samples information and nine physicochemical features. Table S2. Calibration curves of the five saponins. Figure S1. Comparison of 9 physicochemical features among 4-year-old AG cultivated in Jilin, Beijing, and Shanxi. Figure S2. Measure of feature importance. (A–D) The permutation importance of EN, KNN, SVM and MLP. The code is freely available at https://github.com/dreadlesss/Panax_age_predictor. [file 13020_2021_511_MOESM1_ESM.docx]

Additional file 1

Machine Learning Methods to Predict the Cultivation Age of Panacis Quinquefolii Radix

Xiaowen Hu ^1,†^, Hua Yan ^1,†^, Xiaodong Wang ^2^, Zonghu Wang ^2^, Yuanpeng Li ^2^, Lianjun Zheng ^2^, Jianbo Yang ^1^, Wenguang Jing ^1^, Xianlong Cheng ^1^, Feng Wei ^1,^*, Shuangcheng Ma ^1,^*

^1^National Institutes for Food and Drug Control, Institute for Control of Chinese Traditional Medicine and Ethnic Medicine, Beijing 100050, China

^2^XtalPi - AI Research Center (XARC), Tower A, Dongsheng Building, No.8, Zhongguancun East Road, Haidian District, Beijing 100083, China

^†^These authors contributed equally to this work.

Corresponding Authors:

*E-mail address: weifeng@nifdc.org.cn (Feng Wei)

*E-mail address: masc@nifdc.org.cn (Shuangcheng Ma)

Content

[Machine Learning Methods to Predict the Cultivation Age of Panacis Quinquefolii Radix 1](#_Toc81400732)

[Table S1. Samples information and nine physicochemical features. 3](#_Toc81400733)

[Table S2. Calibration curves of the five saponins. 8](#_Toc81400734)

[Figure S1. Comparison of 9 physicochemical features among 4-year-old AG cultivated in Jilin, Beijing, and Shanxi. Data are expressed as mean ± SD. * P < 0.05, ** P < 0.01, *** P < 0.001, **** P < 0.0001, by two tailed Student’s t test. 9](#_Toc81400735)

[Figure S2. Measure of feature importance. (A-D) The permutation importance of EN, KNN, SVM and MLP. 10](#_Toc81400736)

# Table S1. Samples information and nine physicochemical features.

| cultivation region and city | age | approximate length (cm) | weight (g) | ethanol soluble extractives (%) | aqueous soluble extractives (%) | Rg1 (%) | Rd (%) | Re (%) | Rb1 (%) | F11 (%) | data set |
| --- | --- | --- | --- | --- | --- | --- | --- | --- | --- | --- | --- |
| Liaoning, Dandong | 2 | 8.5 | 3.45 | 28.618 | 35.767 | 0.075 | 0.147 | 0.938 | 0.826 | 0.088 | training set |
| Liaoning, Dandong | 2 | 14 | 3.70 | 28.557 | 36.686 | 0.044 | 0.127 | 0.649 | 0.639 | 0.060 | training set |
| Liaoning, Dandong | 2 | 14 | 3.60 | 26.654 | 34.022 | 0.030 | 0.063 | 0.377 | 0.371 | 0.041 | training set |
| Liaoning, Dandong | 2 | 8.5 | 3.15 | 30.172 | 36.732 | 0.029 | 0.043 | 0.279 | 0.235 | 0.032 | training set |
| Liaoning, Dandong | 2 | 8.5 | 3.52 | 29.329 | 37.188 | 0.065 | 0.164 | 0.894 | 0.877 | 0.097 | training set |
| Liaoning, Dandong | 2 | 8.5 | 3.50 | 30.050 | 37.802 | 0.060 | 0.122 | 0.862 | 0.926 | 0.092 | training set |
| Liaoning, Dandong | 2 | 8.5 | 3.47 | 29.000 | 34.792 | 0.035 | 0.123 | 0.562 | 0.616 | 0.067 | training set |
| Liaoning, Dandong | 2 | 14 | 4.18 | 30.260 | 36.248 | 0.039 | 0.089 | 0.517 | 0.512 | 0.049 | training set |
| Liaoning, Dandong | 2 | 5 | 2.04 | 28.608 | 35.714 | 0.074 | 0.129 | 0.750 | 0.651 | 0.090 | training set |
| Liaoning, Dandong | 2 | 5 | 1.91 | 31.089 | 37.866 | 0.036 | 0.035 | 0.294 | 0.234 | 0.033 | training set |
| Liaoning, Dandong | 2 | 8.5 | 3.50 | 30.976 | 38.560 | 0.186 | 0.014 | 0.149 | 0.093 | 0.038 | training set |
| Liaoning, Dandong | 2 | 8.5 | 3.52 | 30.960 | 38.213 | 0.043 | 0.102 | 0.377 | 0.516 | 0.041 | training set |
| Liaoning, Dandong | 2 | 8.5 | 3.14 | 30.827 | 37.209 | 0.070 | 0.158 | 0.765 | 0.844 | 0.069 | training set |
| Liaoning, Dandong | 2 | 6 | 2.81 | 29.083 | 35.847 | 0.106 | 0.012 | 0.183 | 0.098 | 0.030 | training set |
| Liaoning, Dandong | 2 | 14 | 4.65 | 29.857 | 36.501 | 0.177 | 0.118 | 0.583 | 0.463 | 0.048 | training set |
| Liaoning, Dandong | 2 | 6 | 2.60 | 30.951 | 37.292 | 0.062 | 0.106 | 0.592 | 0.614 | 0.053 | training set |
| Liaoning, Dandong | 2 | 14 | 3.71 | 28.903 | 35.337 | 0.025 | 0.064 | 0.315 | 0.370 | 0.032 | training set |
| Liaoning, Dandong | 2 | 5 | 2.50 | 28.526 | 36.366 | 0.022 | 0.045 | 0.220 | 0.197 | 0.024 | training set |
| Liaoning, Dandong | 2 | 6 | 2.75 | 29.187 | 35.923 | 0.132 | 0.116 | 0.692 | 0.632 | 0.070 | training set |
| Liaoning, Dandong | 2 | 5 | 2.51 | 29.180 | 36.598 | 0.050 | 0.072 | 0.519 | 0.412 | 0.052 | training set |
| Liaoning, Dandong | 3 | 12 | 7.77 | 28.578 | 33.259 | 0.069 | 0.217 | 0.932 | 1.316 | 0.089 | training set |
| Liaoning, Dandong | 3 | 8.5 | 7.51 | 33.418 | 38.010 | 0.032 | 0.222 | 0.751 | 1.262 | 0.072 | training set |
| Liaoning, Dandong | 3 | 6 | 6.86 | 31.962 | 36.572 | 0.023 | 0.037 | 0.289 | 0.362 | 0.023 | training set |
| Liaoning, Dandong | 3 | 6 | 3.44 | 32.396 | 35.725 | 0.059 | 0.090 | 0.511 | 0.570 | 0.044 | training set |
| Liaoning, Dandong | 3 | 6 | 4.81 | 35.717 | 36.312 | 0.092 | 0.142 | 0.795 | 1.109 | 0.087 | training set |
| Jilin, Haungwaizi | 3 | 12 | 8.09 | 25.899 | 34.304 | 0.063 | 0.048 | 0.379 | 0.424 | 0.031 | training set |
| Jilin, Zhuxian | 3 | 12 | 8.79 | 34.245 | 35.936 | 0.010 | 0.025 | 0.169 | 0.228 | 0.016 | training set |
| Jilin, Zhuxian | 3 | 8.5 | 5.63 | 28.676 | 30.719 | 0.038 | 0.044 | 0.394 | 0.469 | 0.029 | training set |
| Jilin, Zhuxian | 3 | 12 | 7.11 | 30.634 | 31.780 | 0.088 | 0.195 | 0.896 | 0.806 | 0.064 | training set |
| Jilin, Zhuxian | 3 | 12 | 6.17 | 30.865 | 32.797 | 0.084 | 0.209 | 0.748 | 1.583 | 0.094 | training set |
| Jilin, Zhuxian | 3 | 8.5 | 4.96 | 33.588 | 34.476 | 0.023 | 0.077 | 0.260 | 0.444 | 0.030 | training set |
| Jilin, Zhuxian | 3 | 6 | 4.77 | 32.624 | 33.249 | 0.041 | 0.083 | 0.619 | 0.817 | 0.062 | training set |
| Jilin, Zhuxian | 3 | 12 | 5.66 | 30.714 | 31.090 | 0.014 | 0.032 | 0.117 | 0.193 | 0.012 | training set |
| Jilin, Zhuxian | 3 | 12 | 5.90 | 37.847 | 38.559 | 0.053 | 0.090 | 0.608 | 0.634 | 0.069 | training set |
| Jilin, Zhuxian | 3 | 12 | 5.82 | 30.459 | 33.390 | 0.139 | 0.179 | 0.804 | 1.166 | 0.110 | training set |
| Jilin, Zhuxian | 3 | 6 | 4.60 | 30.368 | 33.292 | 0.089 | 0.187 | 0.763 | 1.358 | 0.069 | training set |
| Jilin, Zhuxian | 3 | 8.5 | 5.39 | 33.719 | 36.586 | 0.045 | 0.129 | 0.672 | 0.835 | 0.059 | training set |
| Jilin, Zhuxian | 3 | 8.5 | 5.05 | 30.065 | 32.518 | 0.014 | 0.019 | 0.127 | 0.121 | 0.094 | training set |
| Jilin, Zhuxian | 3 | 8.5 | 5.49 | 35.373 | 38.712 | 0.008 | 0.036 | 0.151 | 0.232 | 0.172 | training set |
| Jilin, Zhuxian | 3 | 8.5 | 5.40 | 30.600 | 33.282 | 0.030 | 0.037 | 0.188 | 0.303 | 0.016 | training set |
| Shandong, Wendeng | 3 | 8.5 | 5.92 | 48.285 | 48.594 | 0.180 | 0.116 | 1.301 | 2.234 | 0.099 | training set |
| Shandong, Rongcheng | 3 | 6 | 3.72 | 50.512 | 48.130 | 0.024 | 0.054 | 0.297 | 0.636 | 0.036 | training set |
| Shandong, Rongcheng | 3 | 6 | 4.97 | 45.157 | 46.222 | 0.176 | 0.188 | 0.977 | 1.867 | 0.189 | training set |
| Shandong, Rongcheng | 3 | 8.5 | 6.52 | 51.617 | 53.993 | 0.039 | 0.408 | 0.941 | 2.576 | 0.158 | training set |
| Jilin, Haungwaizi | 4 | 8.5 | 14.70 | 32.093 | 34.749 | 0.087 | 0.148 | 0.948 | 1.368 | 0.092 | training set |
| Jilin, Haungwaizi | 4 | 14 | 18.53 | 36.940 | 37.479 | 0.038 | 0.098 | 0.361 | 0.796 | 0.043 | training set |
| Jilin, Haungwaizi | 4 | 8.5 | 13.05 | 28.843 | 30.521 | 0.053 | 0.216 | 0.838 | 1.557 | 0.012 | training set |
| Jilin, Haungwaizi | 4 | 8.5 | 13.14 | 34.185 | 33.422 | 0.065 | 0.254 | 0.737 | 1.834 | 0.120 | training set |
| Jilin, Zhuxian | 4 | 6 | 11.18 | 33.754 | 35.372 | 0.102 | 0.216 | 1.256 | 1.700 | 0.158 | training set |
| Jilin, Zhuxian | 4 | 8.5 | 14.10 | 30.962 | 32.999 | 0.034 | 0.143 | 0.582 | 1.269 | 0.052 | training set |
| Jilin, Zhuxian | 4 | 8.5 | 12.99 | 38.042 | 39.084 | 0.092 | 0.442 | 1.364 | 1.993 | 0.091 | training set |
| Jilin, Zhuxian | 4 | 8.5 | 14.19 | 34.853 | 37.040 | 0.089 | 0.220 | 1.480 | 1.805 | 0.147 | training set |
| Jilin, Zhuxian | 4 | 8.5 | 11.84 | 31.906 | 34.752 | 0.074 | 0.157 | 1.223 | 1.166 | 0.130 | training set |
| Jilin, Zhuxian | 4 | 8.5 | 14.99 | 28.071 | 32.060 | 0.114 | 0.158 | 0.966 | 1.035 | 0.105 | training set |
| Jilin, Xijiang | 4 | 6 | 9.91 | 34.565 | 36.760 | 0.046 | 0.103 | 0.623 | 1.422 | 0.066 | training set |
| Jilin, Xijiang | 4 | 14 | 15.27 | 37.708 | 38.594 | 0.038 | 0.125 | 0.561 | 1.038 | 0.052 | training set |
| Jilin, Xijiang | 4 | 14 | 18.74 | 37.741 | 42.482 | 0.031 | 0.146 | 0.575 | 0.855 | 0.042 | training set |
| Jilin, Xijiang | 4 | 14 | 15.63 | 34.163 | 35.371 | 0.131 | 0.203 | 1.308 | 1.391 | 0.083 | training set |
| Jilin, Dongjiang | 4 | 14 | 21.68 | 39.459 | 41.857 | 0.035 | 0.149 | 0.574 | 0.868 | 0.054 | training set |
| Jilin, Dongjiang | 4 | 14 | 17.49 | 42.135 | 44.226 | 0.033 | 0.099 | 0.525 | 1.067 | 0.070 | training set |
| Jilin, Dongjiang | 4 | 14 | 18.29 | 38.456 | 38.820 | 0.043 | 0.159 | 0.508 | 1.068 | 0.095 | training set |
| Jilin, Dongjiang | 4 | 14 | 17.65 | 45.095 | 43.246 | 0.083 | 0.103 | 0.780 | 1.328 | 0.087 | training set |
| Jilin, Dongjiang | 4 | 14 | 15.32 | 44.996 | 44.559 | 0.096 | 0.086 | 0.357 | 1.086 | 0.056 | training set |
| Jilin, Dongjiang | 4 | 14 | 15.35 | 38.461 | 39.934 | 0.054 | 0.213 | 0.883 | 1.555 | 0.072 | training set |
| Beijing | 4 | 6.40 | 8.56 | 27.664 | 32.887 | 0.064 | 0.045 | 0.564 | 0.591 | 0.063 | test set 1 |
| Beijing | 4 | 6.45 | 9.38 | 27.115 | 30.946 | 0.039 | 0.054 | 0.526 | 0.520 | 0.030 | test set 1 |
| Beijing | 4 | 6.50 | 10.57 | 28.444 | 30.163 | 0.049 | 0.039 | 0.381 | 0.415 | 0.018 | test set 1 |
| Beijing | 4 | 6.55 | 11.23 | 22.772 | 28.031 | 0.028 | 0.049 | 0.346 | 0.572 | 0.019 | test set 1 |
| Beijing | 4 | 6.60 | 15.85 | 25.267 | 30.930 | 0.048 | 0.057 | 0.413 | 0.440 | 0.023 | test set 1 |
| Beijing | 4 | 6.65 | 9.62 | 25.785 | 31.036 | 0.100 | 0.082 | 0.622 | 0.738 | 0.037 | test set 1 |
| Beijing | 4 | 6.70 | 10.83 | 25.563 | 32.256 | 0.187 | 0.093 | 0.901 | 0.824 | 0.057 | test set 1 |
| Beijing | 4 | 6.75 | 11.44 | 24.974 | 28.290 | 0.129 | 0.075 | 0.819 | 0.870 | 0.051 | test set 1 |
| Beijing | 4 | 6.20 | 13.73 | 23.749 | 28.078 | 0.070 | 0.093 | 0.876 | 0.722 | 0.057 | test set 1 |
| Beijing | 4 | 7.30 | 18.74 | 27.664 | 32.887 | 0.064 | 0.045 | 0.564 | 0.591 | 0.063 | test set 1 |
| Beijing | 4 | 7.10 | 17.25 | 27.115 | 30.946 | 0.039 | 0.054 | 0.526 | 0.520 | 0.030 | test set 1 |
| Beijing | 4 | 8.10 | 10.59 | 28.444 | 30.163 | 0.049 | 0.039 | 0.381 | 0.415 | 0.018 | test set 1 |
| Beijing | 4 | 8.50 | 15.48 | 22.772 | 28.031 | 0.028 | 0.049 | 0.346 | 0.572 | 0.019 | test set 1 |
| Beijing | 4 | 9.90 | 16.24 | 25.267 | 30.930 | 0.048 | 0.057 | 0.413 | 0.440 | 0.023 | test set 1 |
| Beijing | 4 | 9.10 | 10.77 | 25.785 | 31.036 | 0.100 | 0.082 | 0.622 | 0.738 | 0.037 | test set 1 |
| Beijing | 4 | 8.30 | 15.21 | 25.563 | 32.256 | 0.187 | 0.093 | 0.901 | 0.824 | 0.057 | test set 1 |
| Beijing | 4 | 7.50 | 14.54 | 23.749 | 28.078 | 0.070 | 0.093 | 0.876 | 0.722 | 0.057 | test set 1 |
| Beijing | 4 | 6.70 | 10.26 | 27.664 | 32.887 | 0.064 | 0.045 | 0.564 | 0.591 | 0.063 | test set 1 |
| Beijing | 4 | 6.75 | 15.37 | 27.115 | 30.946 | 0.039 | 0.054 | 0.526 | 0.520 | 0.030 | test set 1 |
| Beijing | 4 | 6.80 | 12.55 | 28.444 | 30.163 | 0.049 | 0.039 | 0.381 | 0.415 | 0.018 | test set 1 |
| Beijing | 4 | 6.85 | 15.85 | 22.772 | 28.031 | 0.028 | 0.049 | 0.346 | 0.572 | 0.019 | test set 1 |
| Beijing | 4 | 6.90 | 9.62 | 25.267 | 30.930 | 0.048 | 0.057 | 0.413 | 0.440 | 0.023 | test set 1 |
| Beijing | 4 | 6.95 | 10.83 | 25.785 | 31.036 | 0.100 | 0.082 | 0.622 | 0.738 | 0.037 | test set 1 |
| Beijing | 4 | 7.00 | 11.44 | 25.563 | 32.256 | 0.187 | 0.093 | 0.901 | 0.824 | 0.057 | test set 1 |
| Beijing | 4 | 7.05 | 13.73 | 24.974 | 28.290 | 0.129 | 0.075 | 0.819 | 0.870 | 0.051 | test set 1 |
| Shanxi, Liuba | 4 | 2.20 | 1.72 | 46.541 | 51.987 | 0.053 | 0.096 | 0.757 | 1.170 | 0.058 | test set 2 |
| Shanxi, Liuba | 4 | 2.50 | 2.84 | 40.641 | 49.561 | 0.055 | 0.085 | 0.616 | 1.174 | 0.045 | test set 2 |
| Shanxi, Liuba | 4 | 2.50 | 2.41 | 57.226 | 48.491 | 0.134 | 0.133 | 1.041 | 1.176 | 0.091 | test set 2 |
| Shanxi, Liuba | 4 | 2.60 | 4.51 | 47.430 | 45.629 | 0.042 | 0.088 | 0.454 | 1.179 | 0.030 | test set 2 |
| Shanxi, Liuba | 4 | 1.60 | 3.59 | 47.178 | 48.128 | 0.068 | 0.076 | 0.610 | 1.182 | 0.038 | test set 2 |
| Shanxi, Liuba | 4 | 2.80 | 3.26 | 48.173 | 48.252 | 0.081 | 0.102 | 0.799 | 1.186 | 0.050 | test set 2 |
| Shanxi, Liuba | 4 | 2.30 | 4.28 | 51.553 | 51.243 | 0.064 | 0.716 | 0.112 | 1.188 | 0.035 | test set 2 |
| Shanxi, Liuba | 4 | 2.20 | 3.93 | 38.278 | 41.718 | 0.051 | 0.135 | 0.753 | 1.107 | 0.074 | test set 2 |
| Shanxi, Liuba | 4 | 2.90 | 4.68 | 46.541 | 51.987 | 0.053 | 0.096 | 0.757 | 1.170 | 0.058 | test set 2 |
| Shanxi, Liuba | 4 | 3.20 | 4.69 | 40.641 | 49.561 | 0.055 | 0.085 | 0.616 | 1.174 | 0.045 | test set 2 |
| Shanxi, Liuba | 4 | 3.70 | 4.16 | 57.226 | 48.491 | 0.134 | 0.133 | 1.041 | 1.176 | 0.091 | test set 2 |
| Shanxi, Liuba | 4 | 2.90 | 4.45 | 47.430 | 45.629 | 0.042 | 0.088 | 0.454 | 1.179 | 0.030 | test set 2 |
| Shanxi, Liuba | 4 | 4.30 | 6.00 | 47.178 | 48.128 | 0.068 | 0.076 | 0.610 | 1.182 | 0.038 | test set 2 |
| Shanxi, Liuba | 4 | 3.20 | 7.50 | 48.173 | 48.252 | 0.081 | 0.102 | 0.799 | 1.186 | 0.050 | test set 2 |
| Shanxi, Liuba | 4 | 4.40 | 6.30 | 51.553 | 51.243 | 0.064 | 0.716 | 0.112 | 1.188 | 0.035 | test set 2 |
| Shanxi, Liuba | 4 | 3.50 | 8.00 | 38.278 | 41.718 | 0.051 | 0.135 | 0.753 | 1.107 | 0.074 | test set 2 |
| Shanxi, Liuba | 4 | 4.50 | 5.01 | 47.430 | 45.629 | 0.042 | 0.088 | 0.454 | 1.179 | 0.030 | test set 2 |

# Table S2. Calibration curves of the five saponins.

| Saponins | Standard curve | Linearity range (μg) | R^2^ (n=5) | LOD(μg) | LOQ(μg) |
| --- | --- | --- | --- | --- | --- |
| Rg1 | y = 392342x +6561.1 | 0.18-2.77 | 0.998 | 0.18 | 0.46 |
| Re | y = 263361x +48141 | 0.18-2.73 | 0.997 | 0.18 | 0.45 |
| Rb1 | y = 245541x +33618 | 0.22-3.29 | 0.995 | 0.22 | 0.55 |
| Rd | y = 309015x +43573 | 0.20-2.95 | 0.999 | 0.20 | 0.50 |
| F11 | lgy = 1.6378lgx+5.4356 | 0.20-2.92 | 0.998 | 0.20 | 0.48 |

LOD: Limit of detection; LOQ: Limit of quantitation.

# Figure S1. Comparison of 9 physicochemical features among 4-year-old AG cultivated in Jilin, Beijing, and Shanxi. Data are expressed as mean ± SD. * P < 0.05, ** P < 0.01, *** P < 0.001, **** P < 0.0001, by two tailed Student’s t test.

# Figure S2. Measure of feature importance. (A-D) The permutation importance of EN, KNN, SVM and MLP.
